# Livestock-Associated Methicillin-Resistant *Staphylococcus aureus* (MRSA) as Causes of Human Infection and Colonization in Germany

**DOI:** 10.1371/journal.pone.0055040

**Published:** 2013-02-13

**Authors:** Robin Köck, Frieder Schaumburg, Alexander Mellmann, Mahir Köksal, Annette Jurke, Karsten Becker, Alexander W. Friedrich

**Affiliations:** 1 Institute of Hygiene, University Hospital Münster, Münster, Germany; 2 Institute of Medical Microbiology, University Hospital Münster, Münster, Germany; 3 Department of Medical Microbiology, University of Groningen, University Medical Centre Groningen, Groningen, The Netherlands; 4 Department for Infectiology and Hygiene, North Rhine-Westphalian Center for Health, Münster, Germany; Rockefeller University, United States of America

## Abstract

Pigs, cattle and poultry are colonized with MRSA and the zoonotic transmission of such MRSA to humans via direct animal contact, environmental contaminations or meat are a matter of concern. Livestock-associated (LA) MRSA are mostly belonging to clonal complex (CC) 398 as defined by multilocus sequence typing. However, MRSA of other clonal lineages including CC5, CC9 and CC97 have also been detected in livestock animals in Germany. Within the framework of a Dutch-German network project (EUREGIO), 14,036 MRSA isolated from clinical and screening specimens (January 2008 - June 2012) derived from human patients in hospitals as well as general or specialized practices in a German region characterized by a high density of livestock production, were subjected to *S. aureus* protein A (*spa*) sequence typing. The prevalence of putative LA-MRSA among the human MRSA isolates was determined by analyzing the detection of livestock-indicator (LI) *spa* types which had already been reported in German livestock. Overall, 578 *spa* types were detected among the MRSA isolates. LI *spa* types t011, t034, t108, t1451, t2011, t571, t1456, t1250, t1255, t1580, t2970, t2346, t1344, t2576, t2330 and t2510 (all of which are indicative for LA-MRSA CC398) accounted for 18.6% of all human isolates. The LI *spa* types t1430 (CC9), t3992 (CC97), t002 (CC5) and t007 (CC30) were found in 0.14%, 0.01%, 1.01% and 0.04% of all human MRSA isolates, respectively. LI *spa* types associated with CC398 represented 23% of all MRSA from screening samples and a varying proportion among isolates from clinical specimens ranging between 0% in cerebrospinal fluid, 8% in blood cultures and 14% in deep respiratory fluids. Our findings indicate that LA-MRSA are a major cause for human infection and stress the need for close surveillance. Although LA-MRSA CC398 predominates, the occurrence of putative LA-MRSA from other clonal lineages should be monitored.

## Introduction

Methicillin-resistant *Staphylococcus aureus* (MRSA) has been found to colonize livestock including pigs, cattle and poultry. Since many of the MRSA clonal lineages identified in livestock were uncommon for MRSA isolates found until then in human hosts, the term “livestock-associated MRSA” (LA-MRSA) has been introduced to distinguish these MRSA from classical human hospital-acquired (HA-MRSA) or community-associated MRSA (CA-MRSA).

Predominantly, LA-MRSA are associated with clonal complex (CC) 398 as defined by multilocus sequence typing (MLST). Regarding its molecular features, MRSA CC398 represents a distinct subgroup of MRSA mostly characterized by sequence types (ST) 398, ST752 or ST753 [1], harboring the staphylococcal cassette chromosome *mec* (SCC*mec*) types V or IV, accessory gene regulator (agr) type I, capsule type 5 and *S. aureus* protein A gene (*spa*) types t011, t034, t108, t567, t571, t1451, t2011, 2510 and close relatives [2].

In addition to MRSA CC398, other LA-MRSA clonal lineages have also been detected. In a European baseline report, Germany was amongst the countries with the most frequent occurrence of non-CC398 MRSA in pigs. In this report, isolates belonging to the lineages CC9/ST9 (t1430), CC97/ST97 (t3992, t5487) and CC30/ST39 (t007) have been found in 3.9% of all isolates from German pig production [3]. In addition, studies performed in German chicken and turkey identified MRSA associated with the clonal lineages CC9 (t1430) and CC5 (t002) [2], [4]. Isolates from German veal calf belonged to CC9 [5].

In the meantime, epidemiological investigations have shown that LA-MRSA do not only colonize livestock, but are able to overcome the species barrier resulting in zoonotic transmission to persons with direct livestock exposure. Hence, nasal colonization or contamination was found in 23–86% of all pig farmers and veterinarians [6], [7], [8], and in 1–5% of persons with indirect animal exposure (e.g. family members of farmers, farm visitors) [6], [9]. The pathogenicity of MRSA CC398 for humans has been documented in a variety of reports describing cases of endocarditis, otomastoiditis, pneumonia or wound infections [10]. Moreover, MRSA CC398 has been introduced in the healthcare setting mainly in areas with a high density of livestock farming [1], [11]. Although mathematic models indicated that MRSA CC398 were 72% less transmissible in hospitals compared with classical HA-MRSA, the reasons for this difference are unknown [12]. Until now, two healthcare-associated outbreaks of MRSA CC398 have been described in hospitals and nursing homes [13], [14]. A German case-control study showed that a significant proportion (>30%) of patients colonized with MRSA CC398 at admission to a university hospital, had no risk factors indicating an acquisition of the MRSA from farm animals [15]. This was confirmed in a retrospective data analysis of a Dutch hospital where only 11 out of 30 patients colonized with MRSA CC398 had direct contact to livestock [11]. These findings might also indicate that LA-MRSA is also spreading from human to human either in the general population or in healthcare facilities. Regarding the occurrence of LA-MRSA associated with clonal lineages other than CC398 among humans (CC9, CC97), only single cases have hitherto been published [16].

Against this background, there is a need to monitor the occurrence of infections caused by LA-MRSA CC398 and non-CC398 in the healthcare setting. For Germany, only limited data is available regarding this issue [17], [18]. In particular, the occurrence of LA-MRSA other than CC398, has not been assessed in detail until now. Therefore, we investigated the occurrence of MRSA CC398 and other clonal lineages, which have been associated with livestock, among MRSA isolated from clinical and screening specimens derived from human patients treated in German hospitals, and by general practitioners and specialists in ambulatory care participating in a regional preventive network (EurSafety Health-net; www.eursafety.org).

## Methods

The EUREGIO is a part of the Dutch-German border region, which, on the German side of the border, is characterized by a very high density of livestock farming with 530 pigs/km^2^, 71 cattle/km^2^ and 445 chickens/km^2^ [19], respectively.

Since 2005, 39 regional hospitals are cooperating in a network for the prevention of MRSA. In the network, common standards for MRSA admission screening of defined risk–patients at admission (including persons with livestock contact) have been established in 2008 and the hospitals have agreed upon using *spa* typing of selected MRSA isolates for baseline molecular surveillance of MRSA. Five hospitals performed *spa* typing of outbreak-related and bacteraemia isolates only and 34 performed typing of every first MRSA isolate derived from each patient (irrespective of outbreak settings or the specimen the isolate was obtained from). Moreover, MRSA isolates detected from routine microbiological diagnostic specimens derived from outpatients attending 754 general practitioners as well as specialists in the same region, were also *spa* typed. For typing, MRSA isolates were detected in routine microbiology laboratories and forwarded to the central typing facility at the Institute of Hygiene, University Hospital Münster. All typing results were entered in a central database together with information about the isolate’s origin.

For this study, we searched the central database for all MRSA isolates with specified origin typed between 1/1/2008 and 30/6/2012. For identification of MRSA *spa* types in the database, which were indicative for German LA-MRSA clonal lineages, we performed a restriction-free literature search in PubMed (24/7/2012) using the following search terms: Germany AND MRSA AND (livestock or pigs or cattle or poultry or chicken or meat). Moreover, publications of national and European reference institutions were searched for relevant information. Thereby, we found that the following MRSA *spa* types have been detected in German livestock animals or meat samples until now: t011, t034, t108, t145, t571, t1250, t1255, t1344, t1451, t1456, t1580, t1928, t1985, t2011, t2330, t2346, t2510 t2576 and t2970 (all associated with MLST ST398, or single-locus variants within clonal lineage CC398) [2], [3], [4], [15], [20], [21], [22], [23], [24], [25], [26]; t1430 (associated with MLST ST9 and clonal lineage CC9) [2], [3], [5], [25]; t345, t3992, t5487 (associated with MLST ST97 or single-locus variants within clonal lineage CC97) [3], [5], [27]; t002 (associated with MLST ST5 within clonal lineage CC5) [2], [4], [5], [24] and t007 (associated with MLST ST39 within clonal lineage CC30) [3], [5]. These *spa* types were defined as “livestock-indicator” (LI) *spa* types in this study. All human MRSA isolates in the central network database characterized by these LI *spa* types were considered as potential LA-MRSA.

We used the Based Upon repeat pattern (BURP) algorithm of the Ridom StaphType software (Ridom GmbH, Münster, Germany) with default parameters to cluster all *spa* types found in the database into *spa* clonal complexes (*spa*-CC) [28]. The results of the BURP cluster analysis indicate *spa* types which cluster closely with the above defined LI *spa* types. These closely related *spa* types are *spa*-repeat variants and might represent MRSA belonging to the same clonal lineage.

Statistical differences in the proportions of MRSA CC398 on all MRSA isolates typed were tested using Chi Square test for linear trend analysis (EpiInfo version 7, CDC Atlanta, USA) with a p-value <0.05 considered as statistically significant.

## Results

Overall, the network database contained *spa* typing results of 14,036 human MRSA isolates (2,472 in 2008, 3,393 in 2009, 3,341 in 2010, 3,191 in 2011 and 1,639 in the first half-year 2012, respectively). In total, 11,336 isolates (81%) were from hospitalized patients and 2,700 from outpatients attending general practitioners and specialists in outpatient care. The isolates were derived from screening samples (swabs from nose, throat, axilla, groin, anus; n = 9,414), swabs from skin or mucosa without indicated infection (n = 691), superficial wounds samples (n = 2,085), swabs from deep-seated wounds or tissue (n = 331), abscesses (n = 56), sputa (n = 99), deep respiratory secretions (tracheal/bronchial fluid or lavage, n = 346), blood cultures (n = 194), cerebrospinal fluids (n = 7), urine samples (n = 482), catheters (n = 271) and other secretions (e.g. pleural secretions, ascites; n = 70).

The MRSA isolates were associated with 578 different *spa* types, of which t003 (29%), t032 (25%), t011 (10%), t034 (6%), t004, t014, t008 (each 2%) and t022, t020 (each 1%) were the ten most frequently detected. Seventeen isolates (0.1%) were non-typeable using the *spa* typing approach. Among the 14,036 human MRSA isolates, 2771 (19.7%) belonged to the LI *spa* types which had been detected in German livestock animals before according to the literature search performed (table 1). These isolates mainly (18.6%) belonged to LI *spa* types (t011, t034, t108, t571, t1250, t1255, t1451, t1580, t2011, t2330, t2346, t2576, t2970) known to be associated with MLST CC398 from other studies [2], [3], [4], [15], [20], [21], [22], [23], [24], [25]. LI *spa* types indicative for the MLST clonal lineages CC5, CC9, CC97 and CC30 were only rarely found (table 1).

**Table 1:** Putative livestock-associated MRSA among hospital inpatients and ambulatory patients attending general practitioners and specialists in outpatient care.

*spa*-CC/putative MLST CC^1^	Livestock-indicator LI *spa* types (no. of isolates)^2^	% on all human isolates (inpatients/ambulatory patients)
*spa*-CC011/CC398	t011 (1430), t034 (899), t108 (119), t1451 (63), t2011 (26),t571 (13), t1456 (12), t1250 (10), t1255 (10), t1580 (6),t2970 (5), t2346 (5), t1344 (3), t2576 (3), t2330 (2), t2510 (1)	18.57% (19.3%/15.4%)
Singleton/CC30	t007 (1)	0.01% (0.01%/0%)
*spa*-CC003/CC5	t002 (142)	1.01% (0.99%/1.11%)
*spa*-CC 044/CC97	t3992 (1)	0.01% (0.01%/0%)
*spa*-CC1430/CC9	t1430 (20)	0.14% (0.18%/0%)
Non-LA-MRSA	all other *spa* types	80.26% (78.73%/82.70%)

1 associated MLST clonal complex (CC) for the LI *spa* types as described in literature.

2 *spa* types detected in LA-MRSA from German livestock in other studies (number of isolates detected in this study).

The BURP algorithm clustered the isolates into 13 *spa*-CCs; 32 *spa* types were singletons and 43 *spa* types were excluded from clustering, because they comprised less than five *spa*-repeats. Analysis of the clusters revealed that all except three LI *spa* types related to MLST CC398 were grouped into *spa*-CC011. The three remaining LI *spa* types associated with MLST CC398 were excluded in the BURP analysis, because they comprised a too low number of *spa* repeats (t1344, t1456, t2510). LI *spa* types associated with MLST CC5 (t002) were grouped in *spa*-CC003, CC9 (t1430) in *spa*-CC1430, CC30 (t007) as a singleton and CC97 (t3992) in *spa*-CC044, respectively.

The annual distributions of LI *spa* types associated with the predominant *spa*-CC011/CC398 in different clinical and screening specimens from hospital inpatients and outpatients attending general practitioners and specialists are shown in table 2. For these isolates, we observed a significant trend towards an increasing proportion of MRSA CC398 among specimens from screenings (Χ^2^ = 155.5; p<0.001) and superficial wounds (Χ^2^ = 4.4; p = 0.04) between 2008 and 2012. For LI *sp*a types belonging to other *spa*-CCs, *spa* type t1430 was isolated from screenings (n = 17), urine, deep-seated wound/tissue samples and secretions (each n = 1). LI *spa* type t3992 was isolated from a screening sample, t007 from a deep wound sample and t002 from different specimens (screenings n = 81; superficial wounds n = 24; uninfected skin n = 11; deep respiratory secretions n = 9; deep-seated wound/tissue n = 4; catheters and abscesses each n = 3; blood cultures and various secretions each n = 2; sputum and cerebrospinal fluid each n = 1).

**Table 2:** Distribution of MRSA CC398 in different clinical and screening specimens.

Source	2008^1^	2009^1^	2010^1^	2011^1^	2012^1,2^	total^1^
Screening specimens	221/1615 (14%)	417/2221 (19%)	585/2266 (26%)	614/2162 (28%)	334/1150 (29%)	2171/9414 (23%)
Skin/mucosa uninfected	14/115 (12%)	14/160 (9%)	24/184 (13%)	25/164 (15%)	7/68 (10%)	84/691 (12%)
Superficial wounds	29/414 (7%)	48/522 (9%)	48/482 (10%)	57/471 (12%)	18/168 (10%)	200/2075 (10%)
Deep-seated wounds/tissue	1/48 (2%)	14/85 (16%)	5/64 (8%)	13/62 (21%)	2/72 (3%)	25/331 (11%)
Abscesses	1/8 (13%)	0/12 (0%)	0/14 (0%)	3/16 (19%)	0/6 (0%)	4/56 (7%)
Sputum	2/22 (9%)	5/24 (21%)	3/27 (11%)	2/16 (13%)	3/10 (30%)	15/99 (15%)
Deep respiratory fluids	7/58 (12%)	11/110 (10%)	15/68 (22%)	5/62 (8%)	10/48 (21%)	48/346 (14%)
Blood cultures	1/45 (2%)	1/46 (2%)	7/43 (16%)	6/44 (14%)	1/16 (6%)	16/194 (8%)
Cerebrospinal fluid	0/1	0/2	0/0	0/2	0/2	0/7
Urine	3/100 (3%)	2/123 (2%)	4/111 (4%)	7/101 (7%)	2/47 (4%)	18/482 (4%)
Catheters	1/33 (3%)	1/74 (1%)	4/63 (6%)	3/73 (4%)	1/28 (4%)	10/271 (4%)
Other secretions (pleural, ascites, drainage)	0/13 (0%)	0/14 (0%)	1/19 (5%)	4/18 (22%)	1/6 (17%)	6/70 (9%)
Total	280/2472 (11%)	513/3393 (15%)	696/3341 (21%)	739/3191 (23%)	379/1639 (23%)	2607/14036 (19%)

1 number of isolates associated with MRSA CC398/all isolates from the respective specimen typed in the respective period of time (%).

2 first half-year 2012.

Further analysis of the population snapshots of *spa*-CCs 011, 044 and 1430 revealed that several *spa* types detected among the human MRSA isolates showed a *spa* repeat pattern closely related to known LI *spa* types: within *spa*-CC011 this includes 40 *spa* types and 92 (0.7% of all 14,036) MRSA isolates (t567, t588, t898, t1197 (n = 9), t1457 (n = 4), t1606 (n = 2), t1793 (n = 7), t2370 (n = 4), t2741, t2582 (n = 12), t2876 (n = 2), t2971 (n = 2), t3075, t3275, t3423 (n = 2), t3933, t3934, t4030, t4208, t4652 (6), t4677, t4715, t4854, t5095, t5210, t5675, t5883, t6015, t6575, t6606, t6867, t7102, t7336, t7822, t8827, t9013, t9266, t9433, t9796, t10686; all n = 1 except indicated). In both *spa*-CC044 (t224, t359 (n = 2), t1236 (n = 2), t2770, t964; all n = 1 except indicated) and *spa*-CC1430 (t899 (n = 4), t10204, t337, t1419, t100; all n = 1 except indicated) there were five *spa* types clustering closely with LI *spa* types accounting for 0.05% and 0.06% of all 14,036 MRSA isolates included in this study, respectively. LI *spa* type t002 was grouped in *spa*-CC003 and clustered with several closely related *spa* types (e.g. t003) representing typical MRSA *spa* types found among humans.

## Discussion

In November 2006, an MRSA screening of all patients (n = 25,540) admitted to hospitals in the Dutch-German border region EUREGIO revealed, that a significant proportion of MRSA isolates (17% on the German and 89% on the Dutch side of the border) were associated with LA-MRSA CC398 *spa* types [29]. In this study, we have shown that this proportion has further increased to 29% in 2012 in the same hospitals. The high regional admission prevalence of LA-MRSA CC398 can be explained, because the German part of the EUREGIO located in Northwestern Germany is amongst those areas characterized by the highest densities of pig production in Germany and a close association between density of farming and the occurrence of LA-MRSA has been described elsewhere [1]. The increase observed could either be explained by increasing awareness of this risk-factor when performing admission screenings (although all participating hospitals have implemented a common strategy for screening including persons with livestock contact already in 2008) or could be due to increasing introduction of such isolates in the hospitals.

However, our finding that, apart from the detection in screening samples, MRSA CC398 also accounted for a significant proportion of MRSA isolated from clinical specimens including blood cultures (8%) and deep respiratory tract secretions (14%), clearly documents that MRSA CC398 is able to cause severe human infections in hospitals [30]. Since most healthcare-associated *S. aureus* infections are caused by MRSA strains colonizing the host prior to the infection [31], this finding was expected against the background of the high prevalence of this clonal lineage at admission. However, when compared with German national data showing that MRSA CC398 accounts for only 2% of all MRSA from inpatients [18], our findings confirm that MRSA CC398 still has a higher significance in livestock-dense areas [17].

Important questions related to the epidemiology of MRSA CC398 are 1.) whether they lead to an increase of the overall incidence of MRSA colonization or infection among humans and 2.) whether MRSA CC398 are strictly involving persons with direct livestock contact or how often MRSA CC398 is spreading from these persons onwards in the general population. It is a clear limitation of this study that we can neither provide epidemiological data regarding risk factors associated with LA-MRSA carriage, nor data on the overall incidence of MRSA. This is due to the fact that the central network database contains anonymized datasets and not all regional hospitals are typing every first MRSA isolate. Consequently, we cannot estimate for the study setting to what extent LA-MRSA have replaced classical healthcare-associated (HA) MRSA clonal lineages in hospitals or to what extent LA-MRSA have come “on-top” to the overall burden of MRSA cases. The latter has been shown for hospitals in the Netherlands [11]. In addition, it was reported in recent Dutch studies that, although the risk factor “direct livestock contact” is strongly associated with LA-MRSA colonization, the proportion of patients carrying MRSA CC398 without reporting animal contact is reaching 15–37% [11], [32]. As phylogenetic analyses support that most cases of MRSA CC398 among humans are due to the zoonotic transmission from livestock rather than due to livestock-independent *de novo* acquisition of methicillin-resistance genes by methicillin-susceptible *S. aureus* CC398 [33], these findings highlight the question, whether LA-MRSA CC398 is spreading from human-to-human more often than expected.

Besides direct exposition to livestock, potential sources for transmission include farm visits or having a family member employed in farming [6], [9]. Airborne emission of MRSA from livestock farms has been reported, but only in very low concentrations [34]; epidemiological investigations in Germany have not confirmed that persons living in the neighborhood of farms were more likely to be colonized than others [9]. However, living in livestock-dense areas was recently identified as a risk factor for MRSA CC398 carriage in The Netherlands, independently of whether the carrier had direct livestock contact [35]. In addition, meat, which is contaminated frequently with MRSA, could also be a vector for transmission to consumers (via ingestion or handling of uncooked meat). Although the risk for toxin-mediated foodborne disease is generally considered to be low [36], risk assessment for transmission of MRSA is a pending issue [37]. The data obtained in this study can add important information regarding this issue: In Germany, it is known from national surveillance programs that chicken and pig meat at retail was contaminated with MRSA in 42% and 16%, respectively [38]. Among the MRSA detected, MRSA CC398 predominated, but non-CC398 genotypes (mostly CC9/t1430 and CC5/t002) accounted for about 27% of all MRSA isolated from chicken and 10% from pig meat [5]. To our knowledge, this is the first study that assessed the occurrence of LA-MRSA associated with other than the CC398 clonal lineage (including CC5, CC9, CC97) among human MRSA isolates from Germany. While t002 associated with the clonal lineage CC5 is an MRSA clone accounting for about 6% of all (healthcare-associated) MRSA from human hosts in Germany since decades [18], other non-CC398 LA-MRSA (CC9 and CC97) are very rarely found in humans [17], [39]. Changes in the occurrence of these MRSA lineages could therefore indicate changes in the distribution pathways (e.g. food-related transmission). Our data show that a total of 21 isolates characterized by LI *spa* types associated with CC9 and CC97 were found among 14,036 regional isolates typed (0.15%) within a 54-month period. Outpatients were not affected more often than inpatients. It might be argued, that if meat was an important source for acquisition of LA-MRSA in the community, one should not only detect a rising occurrence of MRSA CC398 isolates as described in this study, but also an emergence of non-CC398 clones, such as CC9 or CC97.

Since it has been shown that MRSA *spa* types are rapidly evolving by gaining and losing of *spa* repeats [40] resulting in “new” *spa* types, the assessment of closely related *spa* repeat variants using the BURP algorithm is a feasible tool for detecting types belonging to the same clonal lineage [41]. Indeed, many of the additional *spa* types assigned to *spa*-CC011 in this study (e.g. t1197, t567, t2370, t1457, t1793, t4854) have been identified in livestock animals in other European countries and have been confirmed to belong to the CC398 lineage [3]. This has also been found for isolates within *spa*-CC044 (CC97) [42], [43], [44] and *spa*-CC1430 (CC9) [45], [46]. In consequence, further investigations into the molecular epidemiology of MRSA in German livestock are warranted to confirm whether isolates associated with these *spa* types identified in this study are also linked to livestock.

Overall, we have shown that MRSA CC398 associated with a great variety of different *spa* types accounts for a significant proportion of all cases of MRSA colonization and infection in 39 German hospitals located in a rural area as well as in general and specialized practices in the same region. Moreover, we confirmed that other putative LA-MRSA including t1430/CC9, t3992/CC97, t007/CC30 and t002/CC5 also occur among human MRSA isolates. Since the surveillance of LA-MRSA among humans is mostly focusing on MRSA CC398 until now, our findings stress that it should include also other MRSA clonal lineages potentially associated with livestock.
